# Tracking severe acute respiratory syndrome coronavirus 2 transmission and co‐infection with other acute respiratory pathogens using a sentinel surveillance system in Rift Valley, Kenya

**DOI:** 10.1111/irv.13227

**Published:** 2023-11-29

**Authors:** Vincent Kiplangat Ruttoh, Samwel Lifumo Symekher, Janet Masitsa Majanja, Silvanos Mukunzi Opanda, Esther Wanguche Chitechi, Meshack Wadegu, Ronald Tonui, Peter Kipkemboi Rotich, Tonny Teya Nyandwaro, Anne Wanjiru Mwangi, Ibrahim Ndungu Mwangi, Robert Momanyi Oira, Audrey Gwazima Musimbi, Samson Muuo Nzou

**Affiliations:** ^1^ Centre for Virus Research Kenya Medical Research Institute Nairobi Kenya; ^2^ Department of Molecular Biology and Biotechnology Pan African University Institute of Basic Sciences Technology and Innovation Nairobi Kenya; ^3^ Centre for Microbiology Research Kenya Medical Research Institute Nairobi Kenya; ^4^ Centre for Biotechnology Research and Development Kenya Medical Research Institute Nairobi Kenya

**Keywords:** acute respiratory infections, influenza, SARS‐CoV‐2, sentinel surveillance

## Abstract

**Background:**

The emergence of severe acute respiratory syndrome coronavirus 2 (SARS‐CoV‐2) has been the most significant public health challenge in over a century. SARS‐CoV‐2 has infected over 765 million people worldwide, resulting in over 6.9 million deaths. This study aimed to detect community transmission of SARS‐CoV‐2 and monitor the co‐circulation of SARS‐CoV‐2 with other acute respiratory pathogens in Rift Valley, Kenya.

**Methods:**

We conducted a cross‐sectional active sentinel surveillance for the SARS‐CoV‐2 virus among patients with acute respiratory infections at four sites in Rift Valley from January 2022 to December 2022. One thousand two hundred seventy‐one patients aged between 3 years and 98 years presenting with influenza‐like illness (ILI) were recruited into the study. Nasopharyngeal swab specimens from all study participants were screened using a reverse transcription‐quantitative polymerase chain reaction (RT‐qPCR) for SARS‐CoV‐2, influenza A, influenza B and respiratory syncytial virus (RSV).

**Results:**

The samples that tested positive for influenza A (*n* = 73) and RSV (*n* = 12) were subtyped, while SARS‐CoV‐2 (*n* = 177) positive samples were further screened for 12 viral and seven bacterial respiratory pathogens. We had a prevalence of 13.9% for SARS‐CoV‐2, 5.7% for influenza A, 2% for influenza B and 1% for RSV. Influenza A‐H1pdm09 and RSV B were the most dominant circulating subtypes of influenza A and RSV, respectively. The most common co‐infecting pathogens were *Streptococcus pneumoniae* (*n* = 29) and *Haemophilus influenzae* (*n* = 19), accounting for 16.4% and 10.7% of all the SARS‐CoV‐2 positive samples.

**Conclusions:**

Augmenting syndromic testing in acute respiratory infections (ARIs) surveillance is crucial to inform evidence‐based clinical and public health interventions.

## 1 INTRODUCTION

In late 2019, a cluster of pneumonia cases of unknown aetiology emerged in Wuhan, Hubei Province, China.^1^ A novel coronavirus was reported to be the causative agent and officially named severe acute respiratory syndrome coronavirus 2 (SARS‐CoV‐2) by the International Committee on Taxonomy of Viruses based on phylogenetic analysis.^2^ Within a short period, it had spread to nearly all countries in the world, attaining pandemic status with an estimated global case count of 765 million and 6.9 million deaths as of 24 May 2023.^3^ To date, Kenya has recorded 343,073 SARS‐CoV‐2 cases and 5688 case fatalities.^3^

Acute respiratory infections (ARIs) are a significant public health concern due to their widespread morbidity and mortality and their potential to cause pandemics.^4^, ^5^ ARIs are transmitted primarily via large respiratory droplets, contact with surfaces contaminated by respiratory droplets and aerosolized small respiratory droplets.^6^ Most patients with SARS‐CoV‐2 exhibit fever, sore throat and dry cough, and the less common symptoms are joint aches, rhinorrhoea, myalgia, dizziness, difficulty breathing, diarrhoea, chest pains and nausea.^7^

Early detection and monitoring of ARIs are crucial for controlling outbreaks and preventing their spread. Limited diagnostic capabilities, limited access to health care and economic constraints frustrate early public health interventions. There are over 25 known viral and bacterial ARI pathogens.^8^, ^9^ Patients with ARIs frequently present with symptoms indicative of disease but not specific enough to distinguish what makes them ill clinically. While ARIs are often managed symptomatically, appropriate diagnostic testing and antimicrobial stewardship are essential for optimizing patient care. It is important to note that the presence of bacteria in the upper respiratory tract does not necessarily indicate illness but may reflect asymptomatic carriage.^10^, ^11^ Sentinel surveillance maps the evolution of epidemics and provides evidence to inform control approaches in advance since hospital admissions and mortality indicators lag community transmission.^12^, ^13^ It is an effective tool for monitoring the incidence of ARIs in a population and has been effectively deployed to monitor syndromic illnesses.^14^

Understanding epidemiology and transmission dynamics is vital in providing timely and accurate information for evidence‐based public health interventions. There have been several studies on co‐infection of SARS‐CoV‐2 with other ARIs worldwide, with most focusing on co‐infection with influenza A and B viruses.^15^ There is limited data about SARS‐CoV‐2 co‐infection with other ARI pathogens in Kenya. Viral respiratory infections have been shown to predispose patients to secondary bacterial infections and alter host immunopathology, leading to increased morbidity and mortality.^16^, ^17^ Identifying pathogens co‐infecting with SARS‐CoV‐2 is critical in developing clinical and public health measures to improve patient outcomes. Identifying pathogens co‐infecting with SARS‐CoV‐2 is critical in developing clinical and public health measures that can improve patient outcomes. It is thus prudent to conduct comprehensive syndromic testing for ARI viruses and bacteria, as other co‐infections may go unnoticed, thereby restricting the scope of treatment.

We aimed to address these evidence gaps by conducting an active sentinel surveillance study among patients meeting the case definition of suspected SARS‐CoV‐2 cases. We investigated the incidence of SARS‐CoV‐2, respiratory syncytial virus (RSV), influenza A and influenza B, after which we subtyped influenza A and RSV positive samples. Finally, we investigated the co‐infection of all the SARS‐CoV‐2 positive samples with 12 viral and seven bacterial respiratory pathogens. This manuscript presents the results of an active sentinel surveillance programme conducted in four sentinel sites in the Rift Valley region, Kenya.

## 2 METHODS

### 2.1 Study area

**FIGURE 1:**
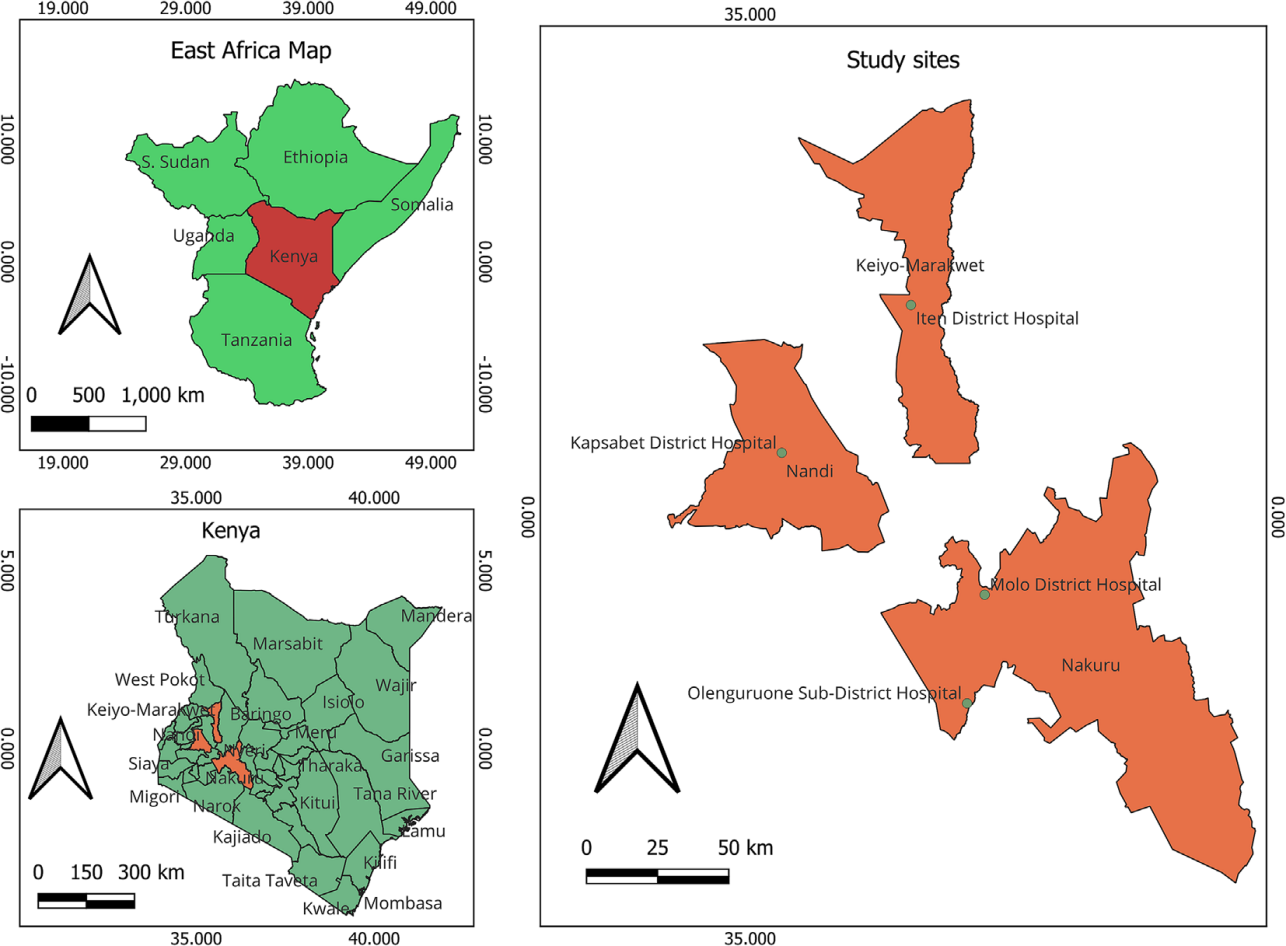
Map showing the location of the study sites in Rift Valley, January 2022–December 2022.

This study was conducted in Nakuru, Elgeyo Marakwet and Nandi Counties in the Rift Valley region. The sites in which the study was implemented include three district hospitals and one sub‐district hospital (Figure 1). The study sites are located in regions of high altitude, situated at elevations ranging from 2000 to 2500 m above sea level and characterized by a temperate climate with low‐temperature ranges typically between 14°C and 25°C. These settings closely mirror the climate and altitude conditions of adjacent counties in Kenya. The study sites were selected purposively due to the paucity of information regarding respiratory infections in these counties and their contributions to the health care burden in Kenya.

### 2.2 Case definition

The study adopted the following case definition for suspected SARS‐CoV‐2 cases: patients with one or more of the following symptoms within the last 7 days: history of fever, cough, sore throat and respiratory distress.

### 2.3 Study design and sample collection

We adopted a cross‐sectional active sentinel surveillance study design. The study population included any person aged 6 months and above. At each sentinel site, the first five patients meeting the case definition of a suspected SARS‐CoV‐2 case were eligible for recruitment per day. Each participant signed a written informed consent form in either English, Kiswahili or the local dialect; for minors, parents or guardians supplied the written consent. Children aged between 13 and 17 were asked to assent to participate in the study after consent had been sought from the caregiver. Socioepidemiological and clinicopathological patient data from each case was recorded using a standardized questionnaire including a unique identifier, demographics, symptoms and pre‐existing conditions onto ODK collect Version 4.4.

### 2.4 Specimen processing

Nasopharyngeal swab specimens were taken using flocked swabs, FLOQswabs (Copan diagnostics, Brescia, Italy), and placed in 2 mL eNAT Viral Transport Medium (VTM) (Copan diagnostics, Brescia, Italy). The specimens were refrigerated at −20°C at the sentinel site and transported in a cold chain within 48 h to the Sample Management and Receiving Facility (SMRF), Kenya Medical Research Institute (KEMRI), where they were stored at −80°C until they were ready for processing.

### 2.5 Detection of SARS‐CoV‐2, RSV and influenza A and B

Prior to extraction, samples were thawed for 30 min and then vortexed for 20 s to ensure a homogenous solution. RNA was extracted using Zymo quick DNA/RNA extraction kit (Zymo Research, Irvine, USA) according to the manufacturer's protocols. Briefly, 400 μL of each sample was extracted and eluted using 50 μL of elution buffer. Reverse transcription‐quantitative polymerase chain reaction (RT‐qPCR) was done using Allplex SARS‐CoV‐2/FluA/FluB/RSV Assay (Seegene Inc, Seoul, South Korea) on a Bio‐Rad CFX96 instrument (Bio‐Rad Laboratories, Hercules, USA). This multiplex assay can simultaneously detect SARS‐CoV‐2 (N gene, RdRP gene and S gene), influenza A, influenza B and RSV. Five microliters of extracted RNA was added to 15 μL of mastermix for each reaction, and amplification was performed at 50°C for 20 min, 95°C for 15 min, 2 cycles of 95°C for 10 s, 60°C for 40 s, 72°C for 20 s, 41 cycles of 95°C for 10 s followed by fluorescence detection at 60°C for 15 s and 72°C for 10 s. All runs were performed together with relevant controls to ensure validity. The results were exported to Microsoft Excel (Office 365) and interpreted using Seegene Viewer (Seegene Inc, Seoul, South Korea).

### 2.6 Influenza A and RSV subtyping

All the influenza A and RSV‐positive samples were subtyped using Seegene Allplex™ respiratory panel 1 (Seegene Inc, Seoul, South Korea). Allplex respiratory panel 1 is a multiplex assay for simultaneous detection and differentiation of three influenza A subtypes (influenza A‐H1, influenza A‐H1pdm09 and influenza A‐H3) and two RSV subtypes (RSV A and RSV B). Viral RNA was extracted from all influenza A positive and RSV positive specimens using Zymo quick DNA/RNA extraction kit (Zymo Research, Irvine, USA) according to the manufacturer's protocol. Eight microliters of extracted RNA was added to 17 μL of mastermix, and RT‐qPCR was performed on a Bio‐Rad CFX96 instrument (Bio‐Rad Laboratories, USA). Amplification was performed at 50°C for 20 min, 95°C for 15 min and 44 cycles of 95°C for 10 s, followed by fluorescence detection at 60°C for 1 min and 72°C for 10 s. All runs were performed together with relevant controls to ensure validity. The results were exported to Microsoft Excel (Office 365) and interpreted using Seegene Viewer (Seegene Inc, Seoul, South Korea).

### 2.7 Detection of other respiratory pathogens

All the positive SARS‐CoV‐2 samples were also analysed for co‐infection with other respiratory pathogens using Seegene Allplex respiratory panels 2, 3 and 4 (Seegene Inc, Seoul, South Korea). These panels are multiplex kits for the identification of 12 viral and seven bacterial pathogens. Respiratory panel 2 identifies adenovirus (AdV), human enterovirus (HEV), human metapneumovirus (hMPV) and parainfluenza virus types 1–4 (PIV 1–4), whereas respiratory panel 3 is for identification of human bocavirus 1/2/3/4 (HBoV 1–4), human coronaviruses 229E (HCoV‐229E), NL63 (HCoV‐NL63), OC43 (HCoV‐OC43) and human rhinovirus (HRV). Respiratory panel 4 is for the identification of respiratory bacterial pathogens *Bordetella parapertussis*, *Bordetella pertussis*, *Chlamydophila pneumoniae*, *Haemophilus influenzae*, *Legionella pneumophila*, *Mycoplasma pneumoniae* and *Streptococcus pneumoniae*. Nucleic acids were extracted from the specimens using Zymo quick DNA/RNA extraction kit (Zymo Research, Irvine, USA) according to the manufacturer's protocol. Eight microliters of extracted nucleic acids was added to 17 μL of mastermix, and RT‐qPCR was performed on a Bio‐Rad CFX96 instrument (Bio‐Rad Laboratories, USA). Amplification was performed at 50°C for 20 min, 95°C for 15 min, 44 cycles of 95°C for 10 s, followed by fluorescence detection at 60°C for 1 min and 72°C for 10 s. The cycling conditions were the same for all the panels. All runs were performed together with relevant controls to ensure validity. The results were exported to Microsoft Excel (Office 365) and interpreted using Seegene Viewer (Seegene Inc, Seoul, South Korea).

### 2.8 Data analysis

The collected data were exported to Microsoft Excel (Office 365) and combined with the results laboratory results. Personal identification data were eliminated prior to statistical analysis. Descriptive statistics were performed for all the variables. Associations between SARS‐CoV‐2 and categorical variables were calculated using the chi‐squared test. All variables with *p*‐values of ≤0.05 were considered statistically significant. The analysis was performed using RStudio (RStudio Inc, Boston, United States).

### 2.9 Ethics statement

This study was approved by the KEMRI Scientific and Ethics Review Unit (SERU No: KEMRI/SERU/CVR/012/4126). All respondents provided informed written consent.

## 3 RESULTS

In the four sentinel sites, 1271 individuals who met the predefined inclusion criteria were enrolled in the study. Both genders were well represented, although there was a slightly higher proportion of females (59.1%, *n* = 761) than males (41.1%, *n* = 510). Most study participants were adults between 19 and 59 years (70.3%, *n* = 894). The median age of the study participants was 37 years, with an age range spanning from 3 to 98 years. SARS‐CoV‐2 was detected in 177 study participants, indicating a prevalence rate of 13.9%, with higher rates observed among females (15.5%, *n* = 118) compared to males (11.6%, *n* = 59). Across different age groups, there was a slight variation in SARS‐CoV‐2 prevalence. The overall prevalence of influenza was 7.6% (*n* = 97), with influenza A being the most common influenza type, with a prevalence of 5.7%, while influenza B was 2%. The majority of those with influenza A were males (7.4%), whereas influenza B exhibited similar rates among males and females. RSV had a prevalence of 1%, with almost similar rates among males and females. There were two instances of co‐infection involving both influenza A and influenza B, one co‐infection with influenza A and RSV and one case where all three pathogens (influenza A, influenza B and RSV) were co‐infecting.

The prevalence of respiratory viruses exhibited notable variations across the different sentinel sites (Table 1). SARS‐CoV‐2 and influenza A and B viruses were found to be circulating in all the sentinel sites, while RSV was present in three sites. Molo had the highest prevalence for both SARS‐CoV‐2 and influenza A, while Kapsabet had the highest rate for RSV. In contrast, Olenguruone had the highest prevalence of influenza B, while Iten consistently had lower prevalence rates across all respiratory viruses.

**TABLE 1:** Demographic characteristics of ARI patients enrolled in the study by gender, age and health facility in Rift Valley region, January 2022–December 2022.

Variables	No. sampled (%)	SARS‐CoV‐2 (%)	RSV (%)	Influenza A (%)	Influenza B (%)
Gender					
Male	510 (40.1)	59 (11.6)	4 (0.8)	38 (7.5)	10 (2.0)
Female	761 (59.9)	118 (15.5)	8 (1.1)	34 (4.5)	15 (2.0)
Age					
≥18	162 (12.8)	22 (13.6)	2 (1.2)	10 (6.2)	5 (3.1)
19–59	894 (70.3)	126 (14.1)	9 (1.0)	54 (6.0)	19 (2.1)
≥60	215 (16.9)	29 (13.5)	1 (0.5)	8 (3.7)	1 (0.5)
Sites					
Iten	479 (37.7)	30 (6.3)	1 (0.2)	5 (1)	1 (0.2)
Olenguruone	430 (33.8)	69 (16.1)	4 (1.0)	27 (6.3)	16 (3.7)
Kapsabet	203 (16.0)	21 (10.3)	7 (3.5)	22 (10.8)	4 (2.0)
Molo	159 (12.5)	57 (35.9)	0 (0)	19 (12.0)	4 (2.5)
Total *n* (%)	1271	177 (13.9)	12 (1.0)	73 (5.7)	25 (2.0)

*Note*: The percentages in column 2 represent column percentages, while the percentages for the subsequent columns represent row percentages.

Abbreviations: ARI, acute respiratory infection; No., number; RSV, respiratory syncytial virus; SARS‐CoV‐2, severe acute respiratory syndrome coronavirus 2.

Among the positive samples for influenza A, the influenza A (H1N1)pdm 2009 subtype accounted for 50.6%. None were identified as influenza A‐H1 or A‐H3, while 49.4% could not be classified into specific subtypes. Fifty percent of the samples tested positive for RSV subtype B, 8.3% (*n* = 1) were identified as RSV subtype A and the rest could not be sub‐typed.

**FIGURE 2:**
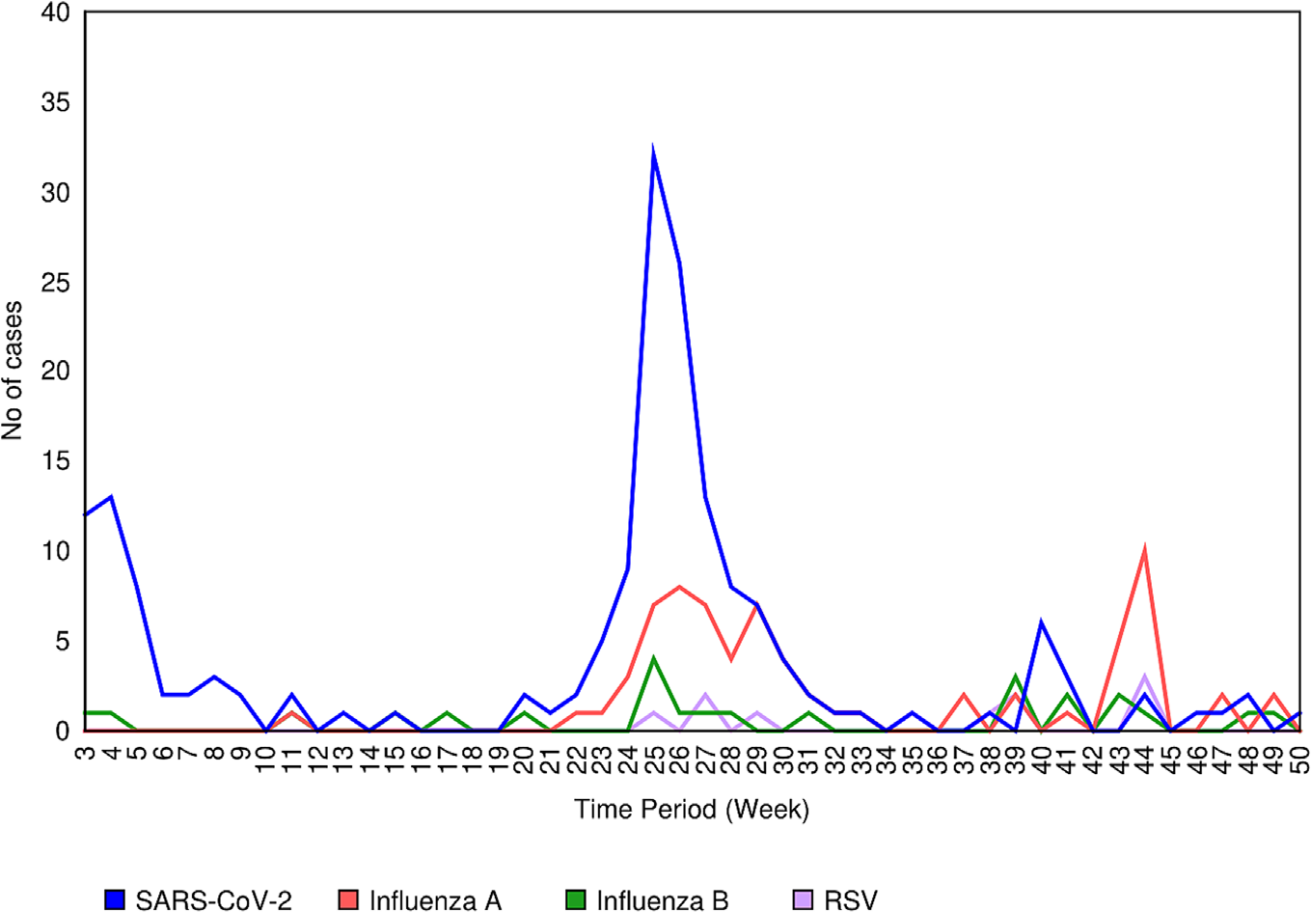
Distribution by epidemiological week of SARS‐CoV‐2, influenza A and B viruses and RSV in Rift Valley region, January 2022–December 2022.

There were two major spikes in the incidence of SARS‐CoV‐2 and influenza A. The spike in SARS‐CoV‐2 occurred in epidemiological weeks 3–9 (January–February) and epidemiological weeks 24–29 (June–July), whereas the spike in influenza A occurred during epidemiological weeks 24–29 (June–July) and epidemiological weeks 36–44 (early September to late October), reflecting the seasonal patterns of influenza occurrence in Kenya (Figure 2).

Among the study participants, clinical symptoms varied across the different aetiologies. Rhinorrhoea (68.4%), myalgia (53.7%), fatigue (54.1%) and fever (52%) were the most prevalent symptoms among the SARS‐CoV‐2 infected patients. The other symptoms were anosmia (24.9%) and diarrhoea (3.9%). Diabetes (2.8%), human immunodeficiency virus (HIV) (1.1%) and chronic respiratory disease (0.6%) were the only reported comorbidities in the SARS‐CoV‐2 positive individuals. A summary of associations between various symptoms and comorbidities with SARS‐CoV‐2 status is shown in Table 2. Statistical analysis revealed a significant association between rhinorrhoea and SARS‐CoV‐2 (OR = 2.463, 95% CI = 1.731–3.523, *p* < 0.001) (Table 2).

**TABLE 2:** Association between symptoms and comorbidities with SARS‐CoV‐2 positivity among patients seeking treatment in the Rift Valley region, January 2022–December 2022.

	SARS‐CoV‐2 negative *n* = 1094 (%)	SARS‐CoV‐2 positive *n* = 177 (%)	OR (CI)	*p*‐value
Fever	506 (46.3)	92 (52)	1.253 (0.901–1.745)	0.168
Anosmia	329 (30)	44 (24.9)	0.772 (0.523–1.123)	0.185
Myalgia	543 (49.6)	95 (53.7)	1.171 (0.841–1.632)	0.332
Rhinorrhoea	510 (46.7)	120(68.4)	2.463 (1.731–3.523)	0.0001***
Fatigue	609 (55.7)	94 (53.1)	0.902 (0.648–1.257)	0.568
Diarrhoea	35 (3.2)	7 (3.9)	1.246 (0.459 to 2.906)	0.648
Chronic respiratory disease	2 (0.6)	1 (0.5)	3.11 (1.806 to 4.935)	0.31
Diabetes	14 (1.2)	5 (2.8)	2.237 (0.622–6.677)	0.1691
HIV	5 (0.5)	2 (1.1)	2.971 (1.760 to 4.926)	0.218

Abbreviations: CI, confidence interval; HIV, human immunodeficiency virus; OR, odds ratio; SARS‐CoV‐2, severe acute respiratory syndrome coronavirus 2.

*** Statistically significant.

Over half of influenza A and B patients presented with rhinorrhoea, fever and myalgia. Both influenza A and B patients also commonly experienced fatigue, with a prevalence of 48.6% and 56%, respectively. Diarrhoea was a less common symptom, with only one case in influenza A and none among influenza B patients. RSV patients mostly presented with myalgia and fever, while the less common symptoms were anosmia, rhinorrhoea and fatigue (Table 3).

**TABLE 3:** Clinical characteristics of influenza A, influenza B and RSV positive patients in Rift Valley region, January 2022–December 2022.

Variables	Influenza A positive *n* = 72 (%)	Influenza B positive *n* = 25 (%)	RSV positive *n* = 12 (%)
Symptoms			
Fever	39 (54.2)	15 (60)	7 (58.3)
Anosmia	20 (27.8)	8 (32)	5 (41.7)
Myalgia	37 (51.4)	14 (56)	9 (75)
Rhinorrhoea	48 (66.7)	19 (76)	4 (33.3)
Fatigue	35 (48.6)	14 (56)	5 (41.7)
Diarrhoea	1 (0.5)	0 (0)	0 (0)
Comorbidities			
Diabetes	1 (0.5)	0 (0)	0 (0)
Exposures			
Smoking	1 (0.5)	0 (0)	0 (0)

Abbreviation: RSV, respiratory syncytial virus.

Approximately a third of the SARS‐CoV‐2 positive individuals were co‐infected with one or more acute respiratory pathogens. Variations in co‐infection rates were observed across the different age groups, with children (0–18 years) having a rate of 31.8% (*n* = 7), while adults (19–59) and older adults (≥ 60) had rates of 31.7% (*n* = 40) and 24.1% (*n* = 7), respectively. The most common co‐infecting pathogens were *Streptococcus pneumoniae*, *Haemophilus influenzae* and HCoV‐OC43. Other co‐infecting pathogens that were detected were HCoV‐229E, influenza A, influenza B, human rhinovirus (HRV) and HCoV‐NL63. RSV, parainfluenza 3 (PIV‐3), parainfluenza 4 (PIV‐4), hMPV, AdV, HEV, HBoV 1‐4, *Bordetella parapertussis*, *Bordetella pertussis*, *Chlamydophila pneumoniae*, *Legionella pneumophila* and *Mycoplasma pneumoniae* were however not detected as co‐infecting with SARS‐CoV‐2 (Table S1).

Seventeen co‐infection patterns with SARS‐CoV‐2 were found in this study (Table 4). There were 31 cases where one pathogen co‐infected with SARS‐CoV‐2, 18 cases of two pathogens, two cases of three pathogens and one case where four pathogens co‐infected with SARS‐CoV‐2. A high proportion of co‐infection patterns was SARS‐CoV‐2 and *Streptococcus pneumoniae*, accounting for 22.6% of all co‐infections observed.

**TABLE 4:** Co‐infection patterns of SARS‐CoV‐2 with other acute respiratory pathogens in Rift Valley region, January 2022 to December 2022.

Virus types	Co‐infecting pathogens	Cases
SARS‐CoV‐2 + influenza A	2	4
SARS‐CoV‐2 + influenza B	2	4
SARS‐CoV‐2 + PIV‐2	2	1
SARS‐CoV‐2 + HCoV‐NL63	2	2
SARS‐CoV‐2 + HCoV‐OC43	2	1
SARS‐CoV‐2 + HRV	2	3
SARS‐CoV‐2 + HI	2	4
SARS‐CoV‐2 + SP	2	12
SARS‐CoV‐2 + PIV‐1 + HI	3	1
SARS‐CoV‐2 + PIV‐1 + HI + SP	4	1
SARS‐CoV‐2 + HCoV‐229E + HI	3	4
SARS‐CoV‐2 + HCoV‐229E + HCoV‐OC43 + HI + SP	5	2
SARS‐CoV‐2 + HCoV‐229E + SP	3	1
SARS‐CoV‐2 + HCoV‐OC43 + SP	3	6
SARS‐CoV‐2 + HRV + SP + HI	4	1
SARS‐CoV‐2 + HI + SP	3	6

Abbreviations: HCoV‐229E, human coronavirus 229E; HCoV‐NL63, human coronavirus NL63; HCoV‐OC43, human coronavirus OC43; HI, *Haemophilus* influenzae; HRV, human rhinovirus; PIV‐1, parainfluenza 1; PIV‐2, parainfluenza 2; SARS‐CoV‐2, severe acute respiratory syndrome coronavirus 2; SP, *Streptococcus pneumoniae*.

## 4 DISCUSSION

The timely detection and monitoring of ARIs is essential for understanding disease patterns and trends, developing appropriate prevention and control strategies and informing public health decision‐making. In this study, we implemented an active sentinel surveillance system for SARS‐CoV‐2 in four sites in the Rift Valley, focusing on detecting SARS‐CoV‐2 and co‐infections with other acute respiratory pathogens. This study offers a glimpse of the respiratory pathogen landscape and the co‐infection of SARS‐CoV‐2 with viral and bacterial pathogens in the Rift Valley, Kenya, providing valuable insights into prevalence and co‐infection patterns.

As of December 2022, Kenya has had seven waves of SARS‐CoV‐2.^18^ The prevalence of SARS‐CoV‐2 infection in our study followed a pattern consistent with the national trend, exhibiting comparable peaks during both the fifth and sixth waves of the Coronavirus Disease 2019 (COVID‐19) pandemic.^18^ This study captures a snapshot of the fifth wave which coincided with the emergence and rapid spread of the Omicron variant of SARS‐CoV‐2. SARS‐CoV‐2 positivity is similar to another study conducted in a geographically analogous region in the Democratic Republic of Congo.^19^

The observed trend of influenza A is consistent with the seasonality of influenza in Kenya, which usually corresponds to the winter season in the Southern hemisphere.^14^ The prevalence of influenza A, influenza B and RSV is much lower than those from previous studies in Kenya.^14^, ^20^ The low prevalence is possibly due to non‐pharmaceutical interventions that were put in place to slow the spread of SARS‐CoV‐2. The dominance in the circulation of influenza A (H1N1)pdm 2009 compared to what has been reported in previous studies suggests a potential change in the dynamics of circulating subtypes, which could impact on the local disease burden.^21^ Whereas the study found a significant association between rhinorrhoea and SARS‐CoV‐2, it is inconsistent with previous studies, which found it to be a rarer symptom of SARS‐CoV‐2.^22^

One of the most significant findings of this study was the high proportion of co‐infections observed in patients with SARS‐CoV‐2 infection, with almost a third of the SARS‐CoV‐2 positive samples being co‐infected with one or more acute respiratory pathogens. *Streptococcus pneumoniae* and *Haemophilus influenzae* were the most co‐infecting ARI pathogens, which is similar to previous studies that identified these two pathogens as some of the most common co‐infecting pathogens in ARIs.^15^, ^23^ Bacterial aetiologies are often not investigated in most ARI cases as they usually present as secondary infections following a viral infection and require further diagnostic approaches, including culturing and antibiotic susceptibility.^24^ While bacterial infections do not necessarily indicate illness, they have been shown to complicate patients' clinical course, leading to poor disease outcomes.^16^ This study's findings thus underscore the importance of diagnosing bacterial ARI pathogens to address challenges arising from co‐infections and prevent unnecessary antibiotic use, which could potentially lead to antimicrobial resistance.^25^

Another notable finding was that human coronaviruses were the common viral aetiologies co‐infected with SARS‐CoV‐2. The circulation of human coronaviruses in Kenya has been reported before the emergence of SARS‐CoV‐2; hence, little is understood about the clinical implications of co‐infection.^26^, ^27^ These findings thus demonstrate the need for syndromic testing for ARIs to aid decision‐making in clinical practice. This highlights the importance of understanding potential interactions and cross‐reactivity between different viruses as this potentially impacts disease severity, immune response, clinical outcomes and therapeutic strategies.^28^, ^29^

The findings from this study are subject to a few limitations. First, it was not possible to make further assessments of the impact of ARIs and co‐infections on patient outcomes due to lack of information on clinical severity, hospitalization, recovery and treatment. Second, our recruitment strategy of recruiting the first five patients meeting the case definition each day may introduce some bias related to the timing of patient presentation. Additionally, recruiting participants exclusively on weekdays could have impacted the diversity of study participants and introduced potential temporal biases. Finally, the study relied on self‐reported symptoms, which may not accurately reflect the clinical presentation of ARIs.

## 5 CONCLUSION

The findings from this study have important implications for public health policies and strategies to reduce the burden of ARIs in Kenya. It is the first study that reveals the co‐infection of SARS‐CoV‐2 with other respiratory pathogens in Kenya, demonstrating that other underlying pathologies warrant syndromic testing for evidence‐based public health interventions to minimize community impact. Sustained surveillance efforts of ARIs are necessary to monitor disease trends and inform public health decision‐making.

## AUTHOR CONTRIBUTIONS

**Vincent Kiplangat Ruttoh:** Conceptualization; project administration; funding acquisition; writing—original draft. **Samwel Lifumo Symekher:** Conceptualization; supervision; funding acquisition; writing—review and editing. **Janet Masitsa Majanja:** Investigation; data curation; writing—review and editing. **Silvanos Mukunzi Opanda:** Investigation; data curation; writing—review and editing. **Esther Chitechi Wanguche:** Investigation; data curation; writing—review and editing. **Meshack Wadegu:** Investigation; methodology; writing—review and editing. **Ronald Tonui:** Formal analysis; software; visualization; writing—original draft. **Peter Kipkemboi Rotich:** Investigation; methodology; writing—review and editing. **Tonny Teya Nyandwaro:** Formal analysis; software; visualization; writing—review and editing. **Anne Wanjiru Mwangi:** Data curation; investigation; supervision. **Ibrahim Ndungu Mwangi:** Conceptualization; funding acquisition; writing—review and editing. **Robert Momanyi Oira:** Investigation; methodology; validation. **Audrey Gwazima Musimbi:** Investigation; data curation; methodology. **Samson Muuo Nzou:** Conceptualization; funding acquisition; supervision; project administration; supervision; writing—original draft.

## CONFLICT OF INTEREST STATEMENT

The authors declare that they have no conflicts of interest.

### PEER REVIEW

The peer review history for this article is available at https://www.webofscience.com/api/gateway/wos/peer-review/10.1111/irv.13227.

## Supporting information


**Table S1:** Distribution of the co‐infecting pathogens in Rift Valley region, January 2022–December 2022.

## Data Availability

The data used to support the findings of this study will be made available on request from the corresponding author.
