# Supplementary material for: Tracking severe acute respiratory syndrome coronavirus 2 transmission and co‐infection with other acute respiratory pathogens using a sentinel surveillance system in Rift Valley, Kenya
Source: Influenza Other Respir Viruses. 2023 Nov 29;17(11):e13227. doi: 10.1111/irv.13227 (PMC10686236; doi:10.1111/irv.13227)
Supplement: Supplementary file 1 — Table S1: Distribution of the co‐infecting pathogens in Rift Valley region, January 2022–December 2022. [file IRV-17-e13227-s001.docx]

Supplementary Table 1: Distribution of the co-infecting pathogens in Rift Valley region, January 2022-December 2022.

| **Co-infecting pathogens** | **Cases, n=177 (%)** |
| --- | --- |
| *Streptococcus pneumoniae* | 29 (16.4) |
| *Haemophilus influenzae* | 19 (10.7) |
| Human coronavirus OC43 | 9 (5.1) |
| Human coronavirus 229E | 7 (4) |
| Human Rhinovirus | 4 (2.2) |
| Influenza A | 4 (2.2) |
| Influenza B | 4 (2.2) |
| Human coronavirus NL63 | 2 (1.1) |
| Parainfluenza virus 1 | 2 (1.1) |
| Parainfluenza virus 2 | 1 (0.6) |
| Respiratory syncytial virus | 0 (0) |
| Parainfluenza virus 3 | 0 (0) |
| Parainfluenza virus 4 | 0 (0) |
| Metapneumovirus | 0 (0) |
| Adenovirus | 0 (0) |
| Enterovirus | 0 (0) |
| Bocavirus 1/2/3/4 | 0 (0) |
| *Bordetella parapertussis* | 0 (0) |
| *Bordetella pertussis* | 0 (0) |
| *Chlamydophila pneumoniae* | 0 (0) |
| *Legionella pneumophila* | 0 (0) |
| *Mycoplasma pneumoniae* | 0 (0) |
